# High blood pressure is associated with increased risk of future fracture, but not vice versa

**DOI:** 10.1038/s41598-024-58691-7

**Published:** 2024-04-05

**Authors:** Xiang-Peng Du, Mei-Liang Zheng, Xin-Chun Yang, Mei-Li Zheng

**Affiliations:** 1 Heart Center and Beijing Key Laboratory of Hypertension, Beijing Chao-Yang Hospital, Capital Medical University, 8# Gong-Ti South Road, Beijing, China; 2 Department of Cardiology, Weihaiwei People’s Hospital, Weihai, Shandong China; 3 Department of Orthopedics, The Second Central Hospital of Baoding, Zhuozhou, Hebei China

**Keywords:** Bone fracture, Hypertension, CHNS, Cardiology, Diseases

## Abstract

The association between high blood pressure and fracture showed obvious discrepancies and were mostly between hypertension with future fracture, but rarely between fracture and incident hypertension. The present study aims to investigate the associations of hypertension with future fracture, and fracture with incident hypertension. We included adult participants from the China Health and Nutrition Survey (CHNS) prospective cohort in 1997–2015 (N = 10,227), 2000–2015 (N = 10,547), 2004–2015 (N = 10,909), and 2006–2015 (N = 11,121) (baseline in 1997, 2000, 2004, 2006 respectively and outcome in 2015). Cox proportional hazards models were used to estimate hazard ratios (HRs) and 95% CIs. In the analysis of the association between hypertension and future fracture, the adjusted HRs (95% CIs) were 1.34 (0.95–1.90) in 1997–2015, 1.40 (1.04–1.88) in 2000–2015, 1.32 (0.98–1.78) in 2004–2015, and 1.38 (1.01–1.88) in 2006–2015. In the analysis of the association between fracture and incident hypertension, the adjusted HRs (95% CIs) were 1.28 (0.96–1.72) in 1997–2015, 1.18 (0.94–1.49) in 2000–2015, 1.12 (0.89–1.40) in 2004–2015, and 1.09 (0.85–1.38) in 2006–2015. The present study showed that hypertension history was associated with increased risk of future fracture, but not vice versa.

## Introduction

Bone fracture and hypertension are both closely related with aging, and linking through the association between osteoporosis and arterial calcification^1^. Fractures in the elderly are mainly caused by osteoporosis (commonly seen in people aged over 50 years^2,3^); arterial calcification, as a very harmful kind of ectopic calcification, may cause arterial stiffening^4^, which was leading to blood pressure increase and hypertension^5,6^. Osteoporosis and arterial stiffening have been proved to be closely associated with each other^7–9^. Osteoporosis and arterial calcification are sharing the common molecular mechanisms, in which calcium is loss from bone tissue and ectopic deposition in the arteries^10,11^. Fracture resulting from osteoporosis and hypertension are supposed to be related with each other, even predictor for each other.

Previous studies of the association between hypertension and fracture showed obvious discrepancies, and were mostly focused on the association between hypertension with future fracture, but rarely on the association between fracture and incident hypertension. In the analysis of the association between hypertension with future fracture, cross-sectional^12,13^ and case-control^14–17^ studies showed significantly positive relationships, however, cohort studies^18,19^ reported non-significant relationships in male or both genders. In the present study, we will evaluate the associations of hypertension with future fracture and fracture with incident hypertension in a natural population from the China Health and Nutrition Survey (CHNS) cohort. CHNS is a prospective study that includes multiple ages and cohorts across nine diverse provinces and ten rounds of surveys between 1989 and 2015. It is a large-scale, longitudinal study in China, and allows the researchers to understand the relationships between diseases and risk factors under normal circumstances^20^.

## Methods

### Study population

**Figure 1:**
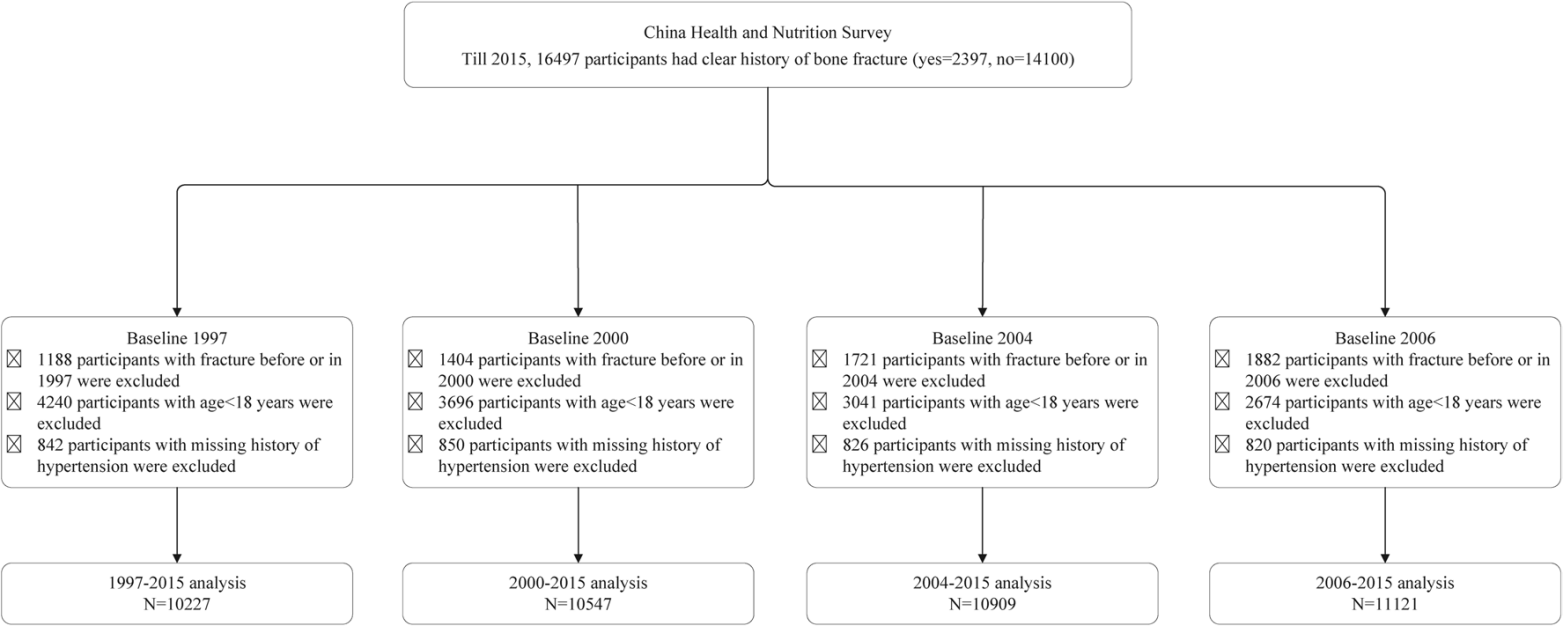
Flow diagram of the analysis of the associations between hypertension and future fracture.

The current study used the longitudinal data from the CHNS. The CHNS is an open, prospective cohort study in China, which have completed 10 rounds of surveys in 1989, 1991, 1993, 1997, 2000, 2004, 2006, 2009, 2011, and 2015. Informed consent was obtained from all subjects. Survey procedures have been described in more detail elsewhere^20–22^. From 1997, the information of bone fracture was recorded, and we analyzed the associations of hypertension with future fracture (Fig. 1), and fracture with incident hypertension (Supplemental Fig. 1) in participants with age ≥ 18 years in 1997–2015 (baseline in 1997 and outcome in 2015), 2000–2015, 2004–2015, and 2006–2015. Inclusion criteria: participants from the CHNS with age ≥ 18 years at baselines, had clear history of hypertension and fracture, and those with missing history of hypertension or fracture were excluded. The research protocol was approved by the Ethics Committee of Beijing Chao-Yang Hospital Affiliated to Capital Medical University in China. The study was carried out in compliance with the Declaration of Helsinki.

### Covariates

Demographic and lifestyle information was obtained through questionnaires, including birth year, gender, diseases history, physical activity, smoking status, and alcohol consumption. Baseline age was calculated as the difference between baseline year and the birth year, and participants with baseline age < 18 years were excluded. Body mass index (BMI) was calculated as weight in kilograms divided by height in meters squared, and body weight and height were measured by well-trained investigators who followed standard measuring procedures. Blood pressure (BP) was measured three times using standard mercury sphygmomanometers by experienced physicians. The average of the three BP measures was used in analyses. Physical activity was estimated from self-reported 7-day recalls of occupational, transportation, domestic, and leisure activities (the International Physical Activity Questionnaires [IPAQ] include the conversion and classification standard of physical activity), and divided into low and high activity level (low < 5000 MET minutes/week, high ≥ 5000 MET minutes/week). Baseline diabetes history, smoking, and alcohol consumption were recorded from self-reported histories.

In the analysis of the associations between BP values and future fracture, we used mean BP values at each baseline (mean values of BP in 1989, 1991, 1993 and 1997 for 1997–2015; mean values of BP in 1989, 1991, 1993, 1997 and 2000 for 2000–2015; mean values of BP in 1989, 1991, 1993, 1997, 2000 and 2004 for 2004–2015; mean values of BP in 1989, 1991, 1993, 1997, 2000, 2004 and 2006 for 2006–2015), and BP included systolic blood pressure (SBP), diastolic blood pressure (DBP), pulse pressure (PP) (PP = SBP-DBP), and mean arterial pressure (MAP) (MAP = DBP + 1/3 PP).The missing values were excluded.

### Outcome

Hypertension was defined as a self-reported diagnosis of hypertension, current use of antihypertensive medication and BP measured of more than 140/90 mmHg. Fracture was defined as a self-reported fracture history. In the analysis of association between hypertension and future fracture, bone fracture was the primary outcome and recorded from the baseline year (not included) till 2015, and a total of 16,497 participants were recorded clear history of fracture till 2015 (Fig. 1); In the analysis of association between fracture and incident hypertension, hypertension was the primary outcome and recorded from the baseline year (not included) till 2015, and a total of 17,499 participants were recorded clear history of hypertension till 2015 (Supplemental Fig. 1).

### Statistical analyses

We conducted all statistical analyses using R statistical software version 4.2.0. Continuous variables with normal distribution were expressed as mean ± standard deviation (SD), and compared by two-sample t-test, while those with non-normal distributed were expressed as quartiles and compared by Mann–Whitney U test. Categorical variables were expressed as percentages and numbers, and compared using the chi-square test. Cox regression models were assessed using the proportional hazards (PH) assumption. Univariable and multivariable Cox regression analyses were used to evaluate the associations of hypertension with future fracture, and fracture with incident hypertension. And we included age, gender, BMI, physical activity, diabetes history, smoking, alcohol consumption in the multivariable model. All statistical tests were two-tailed, and *P*-values of 0.05 were considered to be statistically.

## Results

The present study included two parts: (1) analysis of the association between hypertension and future fracture, and (2) analysis of the association between fracture and incident hypertension. Both of the two parts were carried out in 1997–2015, 2000–2015, 2004–2015, and 2006–2015 (baseline in 1997, 2000, 2004, 2006 and outcome in 2015). The population characteristics in the analysis of the association between hypertension and future fracture was shown in Table 1. In this natural population, male made up nearly half (46.8–47.0%); mean age was increasing as baseline year moving forward (38.4–43.7 years); mean BMI was in an ideal range (23.2–23.5 kg/m^2^); the prevalence of diabetes was relatively low (0.5–1.3%); hypertension prevalence ranged from 1.6 to 5.8%; and the estimated annual incidences of fracture were 547, 524, 483, and 439 cases/per 100,000 people in baseline 1997, 2000, 2004, and 2006, respectively. To show the age distribution, the population was divided into four age categories: < 30, 30–40 (not included), 40–50 (not included), ≥ 50 years, in which participants proportions were close to 1/4 in each category in different baselines. As the baseline year moving forward, participants proportions in the higher age categories were increasing. The population characteristics in the analysis of the association between fracture and incident hypertension was shown in Supplemental Table 1. Demographic and lifestyle information was similar to the above. The estimated annual incidences of hypertension were 1802, 1809, 1728, and 1527 cases/per 100,000 people, respectively.

**Table 1:** Characteristics of the study population in the analysis of hypertension history on future fracture.

	1997–2015 (N = 10,227)	2000–2015 (N = 10,547)	2004–2015 (N = 10,909)	2006–2015 (N = 11,121)
Bone fracture incidence, n (%)	956 (9.3)	795 (7.5)	563 (5.2)	430 (3.9)
Estimated annual incidence of bone fracture, cases/per 100,000 people	547	524	483	439
Bone fracture ≥ 2 times, n (%)	82 (0.8)	67 (0.6)	42 (0.4)	31 (0.3)
Baseline hypertension history, n (%)	165 (1.6)	303 (2.9)	478 (4.4)	642 (5.8)
Baseline male, n (%)	4797 (46.9)	4932 (46.8)	5112 (46.9)	5223 (47.0)
Baseline age, years	38.4 ± 12.4	40.2 ± 12.9	42.7 ± 13.7	43.7 ± 14.2
Baseline age categories, n (%)				
Category 1 < 30 years	2881 (28.2)	2475 (23.5)	2053 (18.8)	2001 (18.0)
30 ≤ Category 2 < 40 years	2742 (26.8)	2891 (27.4)	2670 (24.5)	2469 (22.2)
40 ≤ Category 3 < 50 years	2649 (25.9)	2673 (25.3)	2699 (24.7)	2660 (23.9)
Category 4 ≥ 50 years	1955 (19.1)	2508 (23.8)	3487 (32.0)	3991 (35.9)
Baseline BMI, kg/m^2^	23.2 ± 3.7	23.3 ± 3.7	23.4 ± 3.8	23.5 ± 3.9
Physical activity, n (%)				
Low	7175 (70.2)	7208 (68.3)	5012 (45.9)	4788 (43.1)
High	3052 (29.8)	3339 (31.7)	5897 (54.1)	6333 (56.9)
Baseline diabetes history, n (%)				
Yes	51 (0.5)	84 (0.8)	114 (1.0)	142 (1.3)
No	10,176 (99.5)	10,463 (99.2)	10,795 (99.0)	10,979 (98.7)
Baseline smoking (ever), n (%)				
Yes	2070 (20.2)	2355 (22.3)	2606 (23.9)	2785 (25.0)
No	8157 (79.8)	8192 (77.7)	8303 (76.1)	8336 (75.0)
Baseline alcohol consumption (ever), n (%)				
Yes	2462 (24.1)	2857 (27.1)	3229 (29.6)	3520 (31.7)
No	7765 (75.9)	7690 (72.9)	7680 (70.4)	7601 (68.3)

Baseline data were data in 1997, 2000, 2004, and 2006. BMI, body mass index.

In univariate Cox regression analysis, hypertension history was associated with increased risk of future fracture in 1997–2015 (HR = 2.57, 95% CI 1.83–3.59, *P* < 0.001), 2000–2015 (HR = 2.59, 95% CI 1.96–3.43, *P* < 0.001), 2004–2015 (HR = 2.45, 95% CI 1.86–3.24, *P* < 0.001), and 2006–2015 (HR = 2.36, 95% CI 1.77–3.14, *P* < 0.001). After adjusting for age, the association of hypertension and future fracture seemed to be changed but still of statistical significance in 1997–2015 (HR = 1.47, 95% CI 1.04–2.07, *P* = 0.028), 2000–2015 (HR = 1.54, 95% CI 1.16–2.05, *P* = 0.003), 2004–2015 (HR = 1.40, 95% CI 1.05–1.87, *P* = 0.022), and 2006–2015 (HR = 1.37, 95% CI 1.02–1.85, *P* = 0.039). After adjusting for age, gender, BMI, physical activity, hypertension, diabetes, smoking and alcohol consumption, the association between hypertension and future fracture was still significant in 2000–2015 (HR = 1.40, 95% CI 1.04–1.88, *P* = 0.024) and 2006–2015 (HR = 1.38, 95% CI 1.01–1.88, *P* = 0.045), and not significant in 1997–2015 (HR = 1.34, 95% CI 0.95–1.90, *P* = 0.097) and 2004–2015 (HR = 1.32, 95% CI 0.98–1.78, *P* = 0.071) (Table 2). In multivariate Cox regression analysis, aging, female, high physical activity, smoking and alcohol consumption were not significantly associated with the risk of future fracture (Supplemental Table 2).

**Table 2:** Univariate and multivariate Cox regression analysis of hypertension history on future fracture.

	1997–2015 (Total = 10,227, Cases = 956)			2000–2015 (Total = 10,547, Cases = 795)			2004–2015 (Total = 10,909, Cases = 563)			2006–2015 (Total = 11,121, Cases = 430)		
	HR	95%CI	P value	HR	95%CI	P value	HR	95%CI	P value	HR	95%CI	P value
Crude	2.57	1.83–3.59	< 0.001	2.59	1.96–3.43	< 0.001	2.45	1.86–3.24	< 0.001	2.36	1.77–3.14	< 0.001
Adjusted for age	1.47	1.04–2.07	0.028	1.54	1.16–2.05	0.003	1.40	1.05–1.87	0.022	1.37	1.02–1.85	0.039
Adjusted for age, gender	1.47	1.04–2.07	0.028	1.54	1.16–2.05	0.003	1.40	1.05–1.86	0.023	1.37	1.01–1.85	0.041
Adjusted for age, gender, BMI	1.47	1.04–2.07	0.029	1.53	1.15–2.04	0.004	1.42	1.06–1.90	0.019	1.41	1.04–1.91	0.028
Adjusted for age, gender, BMI, physical activity	1.46	1.03–2.06	0.032	1.52	1.14–2.02	0.005	1.42	1.06–1.90	0.019	1.40	1.03–1.90	0.030
Adjusted for age, gender, BMI, physical activity, diabetes	1.44	1.02–2.03	0.038	1.49	1.11–1.99	0.008	1.40	1.04–1.88	0.026	1.41	1.03–1.92	0.031
Adjusted for age, gender, BMI, physical activity, diabetes, smoking	1.38	0.98–1.95	0.066	1.44	1.07–1.93	0.015	1.35	1.002–1.82	0.048	1.39	1.02–1.90	0.038
Adjusted for age, gender, BMI, physical activity, diabetes, smoking, alcohol consumption	1.34	0.95–1.90	0.097	1.40	1.04–1.88	0.024	1.32	0.98–1.78	0.071	1.38	1.01–1.88	0.045

The present analysis included the associations of hypertension history on future fracture in 1997–2015 (baseline in 1997 and outcome in 2015), 2000–2015, 2004–2015, and 2006–2015. BMI, body mass index.

In the analysis of the associations between BP values and future fracture, we used mean values of BP in 1989, 1991, 1993 and 1997 for baseline 1997, mean values of BP in 1989, 1991, 1993, 1997 and 2000 for baseline 2000, mean values of BP in 1989, 1991, 1993, 1997, 2000 and 2004 for baseline 2004, and mean values of BP in 1989, 1991, 1993, 1997, 2000, 2004 and 2006 for baseline 2006. The missing BP values were excluded. In univariate Cox regression analysis, increase of mean SBP values were associated with increased risk of future fracture in 1997–2015 (every 10 mmHg increase of SBP, HR = 1.19, 95% CI 1.12–1.26, *P* < 0.001), 2000–2015 (every 10 mmHg increase of SBP, HR = 1.23, 95% CI 1.16–1.31, *P* < 0.001), 2004–2015 (every 10 mmHg increase of SBP, HR = 1.17, 95% CI 1.10–1.25, *P* < 0.001), and 2006–2015 (every 10 mmHg increase of SBP, HR = 1.21, 95% CI 1.12–1.30, *P* < 0.001). After multivariate adjusted, the association between mean SBP values and future fracture was still significant in 1997–2015 (every 10 mmHg increase of SBP, HR = 1.07, 95% CI 1.01–1.14, *P* = 0.032) and 2000–2015 (every 10 mmHg increase of SBP, HR = 1.08, 95% CI 1.01–1.16, *P* = 0.024), and not significant in 2004–2015 and 2006–2015. Mean DBP and MAP increases were also associated with increased risk of future fracture in univariate Cox regression analysis, but the association became non-significant in multivariate regression. In univariate Cox regression analysis, increase of mean PP values were associated with increased risk of future fracture in 1997–2015 (every 5 mmHg increase of PP, HR = 1.14, 95% CI 1.08–1.19, *P* < 0.001), 2000–2015 (every 5 mmHg increase of PP, HR = 1.17, 95% CI 1.12–1.23, *P* < 0.001), 2004–2015 (every 5 mmHg increase of PP, HR = 1.13, 95% CI 1.07–1.19, *P* < 0.001), and 2006–2015 (every 5 mmHg increase of PP, HR = 1.17, 95% CI 1.11–1.24, *P* < 0.001). After multivariate adjusted, the association between mean PP values and future fracture was still significant in 1997–2015 (every 5 mmHg increase of PP, HR = 1.06, 95% CI 1.01–1.12, *P* = 0.014), 2000–2015 (every 5 mmHg increase of PP, HR = 1.08, 95% CI 1.02–1.13, *P* = 0.006), and 2006–2015 (every 5 mmHg increase of PP, HR = 1.08, 95% CI 1.01–1.16, *P* = 0.022), and not significant in 2004–2015 (Table 3).

**Table 3:** Univariate and multivariate Cox regression analysis of blood pressure on future fracture.

	1997–2015 (Total = 4574, Cases = 554)			2000–2015 (Total = 5186, Cases = 501)			2004–2015 (Total = 5794, Cases = 369)			2006–2015 (Total = 6163, Cases = 278)		
	HR	95% CI	P value	HR	95% CI	P value	HR	95% CI	P value	HR	95% CI	P value
SBPm, every 10 mmHg												
Crude	1.19	1.12–1.26	< 0.001	1.23	1.16–1.31	< 0.001	1.17	1.10–1.25	< 0.001	1.21	1.12–1.30	< 0.001
Multivariate adjusted*	1.07	1.01–1.14	0.032	1.08	1.01–1.16	0.024	1.04	0.96–1.12	0.341	1.07	0.98–1.18	0.130
DBPm, every 5 mmHg												
Crude	1.10	1.05–1.15	< 0.001	1.12	1.07–1.18	< 0.001	1.10	1.04–1.16	0.002	1.10	1.02–1.17	0.009
Multivariate adjusted*	1.02	0.97–1.08	0.383	1.02	0.97–1.08	0.438	1.01	0.95–1.08	0.770	1.001	0.92–1.08	0.981
PPm, every 5 mmHg												
Crude	1.14	1.08–1.19	< 0.001	1.17	1.12–1.23	< 0.001	1.13	1.07–1.19	< 0.001	1.17	1.11–1.24	< 0.001
Multivariate adjusted*	1.06	1.01–1.12	0.014	1.08	1.02–1.13	0.006	1.04	0.98–1.10	0.221	1.08	1.01–1.16	0.022
MAPm, every 5 mmHg												
Crude	1.11	1.06–1.15	< 0.001	1.14	1.09–1.19	< 0.001	1.10	1.05–1.16	< 0.001	1.12	1.05–1.18	< 0.001
Multivariate adjusted*	1.04	0.99–1.08	0.131	1.04	0.99–1.09	0.130	1.02	0.96–1.07	0.534	1.03	0.96–1.10	0.460

*Multivariate adjusted for gender, age categories, body mass index, physical activity, diabetes history, smoking, alcohol consumption. SBPm, mean values of systolic blood pressure (SBP) before and at baselines (mean values of SBP in 1989, 1991, 1993 and 1997 for 1997–2015; mean values of SBP in 1989, 1991, 1993, 1997 and 2000 for 2000–2015; mean values of SBP in 1989, 1991, 1993, 1997, 2000 and 2004 for 2004–2015; mean values of SBP in 1989, 1991, 1993, 1997, 2000, 2004 and 2006 for 2006–2015); DBPm, mean values of diastolic blood pressure (DBP) before and at baselines; PPm, mean values of pulse pressure (PP) before and at baselines; MAPm, mean values of mean arterial pressure (MAP) before and at baselines.

In the analysis of association between fracture and incident hypertension, bone fracture history was associated with increased risk of incident hypertension in univariate Cox regression analysis (for 1997–2015, HR = 1.97, 95% CI 1.47–2.64, *P* < 0.001; for 2000–2015, HR = 1.68, 95% CI 1.35–2.11, *P* < 0.001; for 2004–2015, HR = 1.47, 95% CI 1.18–1.84, *P* < 0.001; for 2006–2015, HR = 1.48, 95% CI 1.17–1.87, *P* < 0.001), but the association became non-significant after adjusting for age only, and still non-significant in multivariate regression (Table 4).

**Table 4:** Univariate and multivariate Cox regression analysis of bone fracture on incident hypertension.

	1997–2015 (Total = 11,428, Cases = 3181)			2000–2015 (Total = 11,494, Cases = 2725)			2004–2015 (Total = 11,331, Cases = 1946)			2006–2015 (Total = 11,178, Cases = 1428)		
	HR	95%CI	P value	HR	95%CI	P value	HR	95%CI	P value	HR	95%CI	P value
Crude	1.97	1.47–2.64	< 0.001	1.68	1.35–2.11	< 0.001	1.47	1.18–1.84	< 0.001	1.48	1.17–1.87	< 0.001
Adjusted for age	1.30	0.97–1.74	0.080	1.20	0.95–1.50	0.122	**1.10**	0.88–1.38	0.413	1.05	0.83–1.33	0.678
Adjusted for age, gender	1.30	0.97–1.73	0.083	1.19	0.95–1.49	0.129	1.09	0.87–1.37	0.444	1.05	0.83–1.33	0.696
Adjusted for age, gender, BMI	1.34	0.999–1.79	0.050	1.21	0.97–1.52	0.093	1.12	0.89–1.40	0.332	1.08	0.85–1.36	0.531
Adjusted for age, gender, BMI, physical activity	1.33	0.996–1.79	0.053	1.21	0.97–1.52	0.097	1.12	0.90–1.41	0.310	1.08	0.86–1.37	0.509
Adjusted for age, gender, BMI, physical activity, diabetes	1.36	1.01–1.82	0.041	1.23	0.98–1.55	0.069	1.14	0.91–1.43	0.247	1.10	0.87–1.39	0.430
Adjusted for age, gender, BMI, physical activity, diabetes, smoking	1.31	0.98–1.76	0.071	1.20	0.95–1.50	0.120	1.12	0.89–1.40	0.335	1.09	0.86–1.38	0.491
Adjusted for age, gender, BMI, physical activity, diabetes, smoking, alcohol consumption	1.28	0.96–1.72	0.094	1.18	0.94–1.49	0.145	1.12	0.89–1.40	0.337	1.09	0.85–1.38	0.502

The present analysis included the associations of bone fracture on incident hypertension in 1997–2015 (baseline in 1997 and outcome in 2015), 2000–2015, 2004–2015, and 2006–2015. BMI, body mass index.

## Discussion

The present study has an intriguing finding that hypertension history was associated with increased risk of future fracture, but fracture was not significantly associated with increased risk of incident hypertension after adjusting for age only, and still non-significant in the multivariate regression.

**Figure 2:**
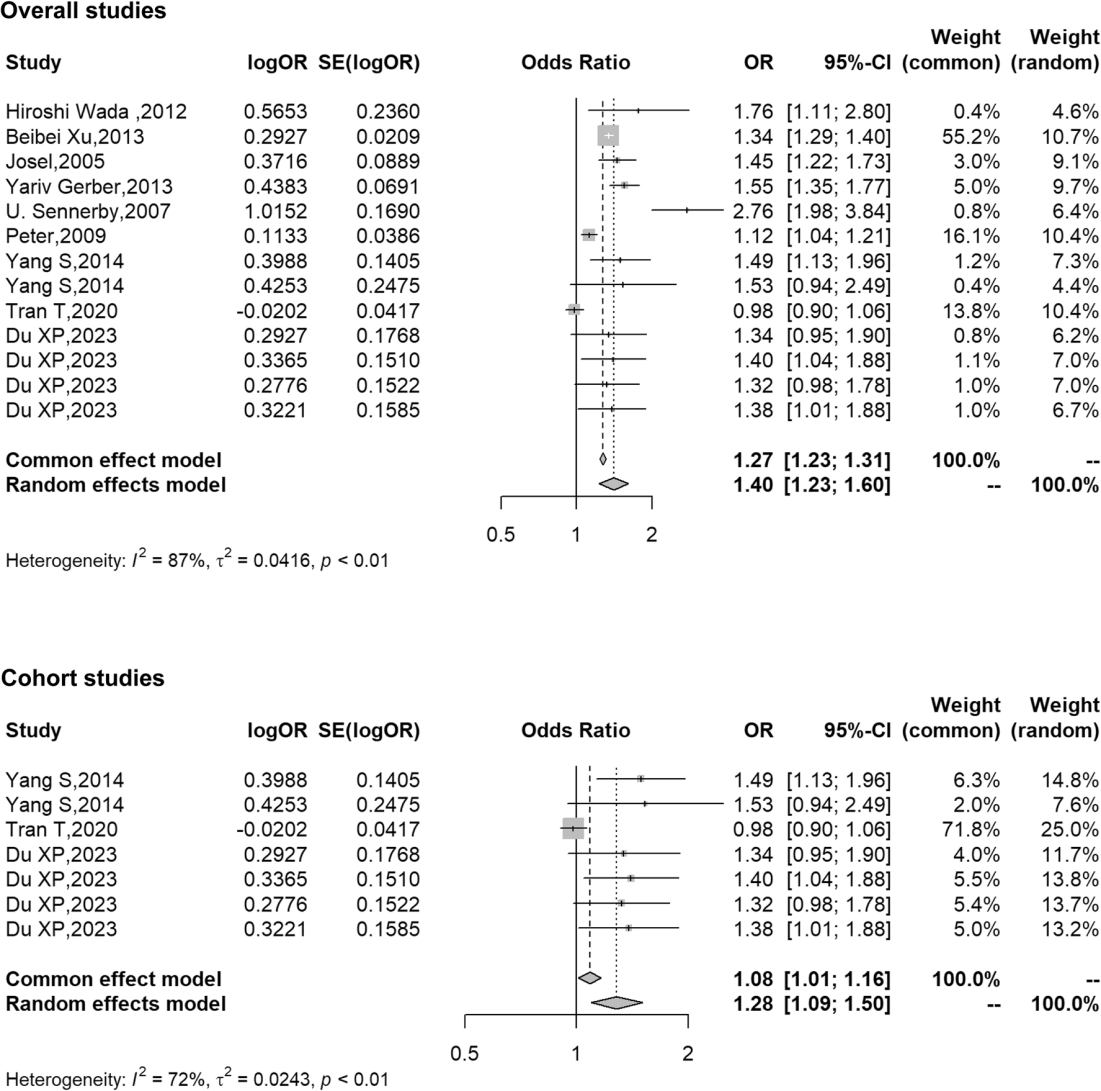
Forest plots of hypertension and future fracture risk in the meta-analysis of overall studies and cohort studies only.

In the studies of association between hypertension and fracture risk, cross-sectional studies of health check-up populations and hospitalized patients reported that hypertension was associated with increased risk of fracture^12,13^. Several case–control studies, by tracing cardiovascular diseases hospitalization prior to fracture, showed that hypertension was associated with increased risk of fracture^14–16^. However, a case–control study^17^ with a small sample size reported hypertension was associated with increased risk of hip fracture only in women but not in men. A prospective cohort study^18^ with a small sample size also showed hypertension was associated with increased risk of hip fracture only in women but not in men. Another cohort study^19^ showed hypertension was not associated with future fracture risk. Obvious discrepancies exist in the reported finding above, so we carried out a systematic review of the published articles, and the results showed hypertension was associated with increased risk of future fracture in overall studies (pooled OR = 1.40, 95% CI 1.23–1.60; *I*^*2*^ = 72%, *P* < 0.01) and cohort studies (pooled OR = 1.28, 95% CI 1.09–1.50; *I*^*2*^ = 87%, *P* < 0.01) (Fig. 2). In the research of association between fracture and hypertension risk, a retrospective case–control study from the National Health and Nutrition Examination Survey showed that fracture was associated with increased risk of hypertension in the crude model, but became non-significant after multivariable adjusted, which is consistent with the present study. The analysis of the present study and previous studies showed that hypertension was associated with increased risk of future fracture, but not vice versa.

Bone fracture and hypertension are diseases both related with aging. Osteoporotic fracture is an influential health issue in the elderly, which will result in substantial bone-associated morbidities and increased mortality^2^. In fact, osteoporosis is commonly detected by X-ray-based imaging in people aged over 50 years^3^. Fractures in the young are more likely to be non-osteoporotic fractures, however, chronic kidney diseases and endocrine abnormalities will result in osteoporosis in the young^23,24^. Hypertension is also an age-related disease. A certain number of studies^6,9^ have discussed the association between hypertension and arterial stiffening, that arterial stiffening may antedate and contribute to the development of hypertension^25^. Arterial medial calcification is the pathophysiological mechanism of arterial stiffening. Generalized arterial calcification of infancy (GACI) will present hypertension after birth^26^. Patients with hypertension are supposed to have arterial calcification, which may or may not be detected. Arterial calcification is a very harmful kind of ectopic calcification, which is sharing the common molecular mechanisms with osteoporosis^11^. In osteoporosis and arterial calcification, calcium is loss from bone tissue and ectopic deposition in the arteries. Based on the results of the present study, we supposed that, when arterial stiffening is leading to hypertension, bone tissue may already have osteoporotic changes, which may or may not be detected. In the follow-up, osteoporosis causes fragility fractures. That is why hypertension may predict future fracture. But on the other hand, fractures are not all osteoporosis-caused. Osteoporosis occurs as arterial calcification happens. Osteoporosis has been proved to be associated with hypertension risk^27^. But fracture may not predict incident hypertension.

The present study has some limitations: first, there are no bone mineral density or other biomarker to evaluate osteoporosis in CHNS, thus, it is difficult to show the effect of osteoporosis in the association between hypertension and fracture, and we can only speculate it from the results of other studies; second, in this natural population, fracture incidence is too low for subgroup analysis, and we can’t perform analysis in different ages or other groups.

### Supplementary Information

Supplementary Legends.

Supplementary Figure 1.

Supplementary Table 1.

Supplementary Table 2.

## Data Availability

The datasets used during the current study available from the corresponding author on reasonable request.
