# Supplementary material for: High blood pressure is associated with increased risk of future fracture, but not vice versa
Source: Sci Rep. 2024 Apr 5;14:8005. doi: 10.1038/s41598-024-58691-7 (PMC10997641; doi:10.1038/s41598-024-58691-7)
Supplement: Supplementary file 1 — Supplementary Legends. [file 41598_2024_58691_MOESM1_ESM.docx]

Supplemental Figure 1. Flow diagram of the analysis of the associations between fracture and incident hypertension
