# Supplementary figures and images for: High blood pressure is associated with increased risk of future fracture, but not vice versa
Source: Sci Rep. 2024 Apr 5;14:8005. doi: 10.1038/s41598-024-58691-7 (PMC10997641; doi:10.1038/s41598-024-58691-7)

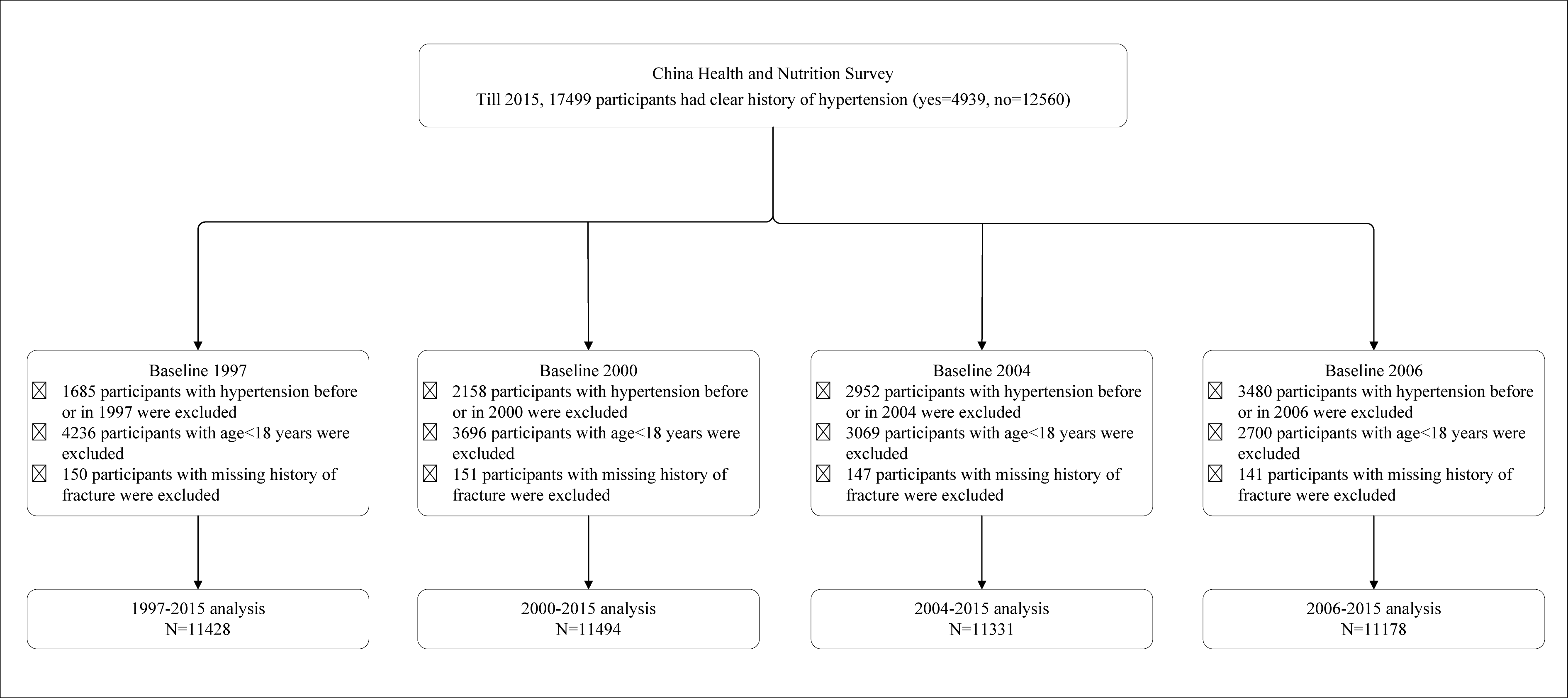

Supplement: Supplementary file 2 — Supplementary Figure 1. [file 41598_2024_58691_MOESM2_ESM.tif]
