# Supplementary material for: High blood pressure is associated with increased risk of future fracture, but not vice versa
Source: Sci Rep. 2024 Apr 5;14:8005. doi: 10.1038/s41598-024-58691-7 (PMC10997641; doi:10.1038/s41598-024-58691-7)
Supplement: Supplementary file 3 — Supplementary Table 1. [file 41598_2024_58691_MOESM3_ESM.docx]

Supplemental Table 1. Characteristics of the study population in the analysis of bone fracture on incident hypertension

|  | 1997-2015 (N=11428) | 2000-2015  (N=11494) | 2004-2015 (N=11331) | 2006-2015  (N=11178) |
| --- | --- | --- | --- | --- |
| Hypertension incidence, n (%) | 3181 (27.8) | 2725 (23.7) | 1946 (17.2) | 1428 (12.8) |
| Estimated annual incidence of hypertension, cases/per 100,000 people | 1802 | 1809 | 1728 | 1527 |
| Baseline bone fracture, n (%) | 100 (0.9) | 220 (1.9) | 337 (3.0) | 416 (3.7) |
| Baseline male, n (%) | 5452 (47.7) | 5473 (47.6) | 5416 (47.8) | 5344 (47.8) |
| Baseline age, years | 39.2±12.5 | 40.6±12.9 | 42.4±13.4 | 43.0±13.8 |
| Baseline age categories, n (%) |  |  |  |  |
| Category 1<30 years | 2989 (26.2) | 2556 (22.2) | 2117 (18.7) | 2071 (18.5) |
| 30≤Category 2<40 years | 2987 (26.1) | 3108 (27.1) | 2824 (24.9) | 2583 (23.1) |
| 40≤Category 3<50 years | 3058 (26.8) | 3003 (26.1) | 2899 (25.6) | 2773 (24.8) |
| Category 4≥50 years | 2394 (20.9) | 2827 (24.6) | 3491 (30.8) | 3751 (33.6) |
| Baseline BMI, kg/m^2^ | 23.3±3.9 | 23.4±3.9 | 23.4±4.0 | 23.4±4.0 |
| Physical activity, n (%) |  |  |  |  |
| Low | 8102 (70.9) | 7883 (68.6) | 5246 (46.3) | 4798 (42.9) |
| High | 3326 (29.1) | 3611 (31.4) | 6085 (53.7) | 6380 (57.1) |
| Baseline diabetes history, n (%) |  |  |  |  |
| Yes | 51 (0.4) | 76 (0.7) | 79 (0.7) | 79 (0.7) |
| No | 11377 (99.6) | 11418 (99.3) | 11252 (99.3) | 11099 (99.3) |
| Baseline smoking (ever), n (%) |  |  |  |  |
| Yes | 2328 (20.4) | 2564 (22.3) | 2679 (23.6) | 2744 (24.5) |
| No | 9100 (79.6) | 8930 (77.7) | 8652 (76.4) | 8434 (75.5) |
| Baseline alcohol consumption (ever), n (%) |  |  |  |  |
| Yes | 2740 (24.0) | 3081 (26.8) | 3268 (28.8) | 3444 (30.8) |
| No | 8688 (76.0) | 8413 (73.2) | 8063 (71.2) | 7734 (69.2) |

Baseline data were data in 1997, 2000, 2004, and 2006. BMI, body mass index.
