# Supplementary material for: High blood pressure is associated with increased risk of future fracture, but not vice versa
Source: Sci Rep. 2024 Apr 5;14:8005. doi: 10.1038/s41598-024-58691-7 (PMC10997641; doi:10.1038/s41598-024-58691-7)
Supplement: Supplementary file 4 — Supplementary Table 2. [file 41598_2024_58691_MOESM4_ESM.docx]

Supplemental Table 2. Multivariate COX regression analysis of covariates on future fracture

|  | 1997-2015 | | |  | 2000-2015 | | |  | 2004-2015 | | |  | 2006-2015 | | |
| --- | --- | --- | --- | --- | --- | --- | --- | --- | --- | --- | --- | --- | --- | --- | --- |
|  | HR | 95%CI | P value |  | HR | 95%CI | P value |  | HR | 95%CI | P value |  | HR | 95%CI | P value |
| Hypertension history | 1.34 | 0.95-1.90 | 0.097 |  | 1.40 | 1.04-1.88 | 0.024 |  | 1.32 | 0.98-1.78 | 0.071 |  | 1.38 | 1.01-1.88 | 0.045 |
| Female | 1.22 | 1.03-1.45 | 0.022 |  | 1.20 | 0.99-1.45 | 0.061 |  | 1.23 | 0.98-1.55 | 0.072 |  | 1.31 | 1.003-1.71 | 0.048 |
| Age | 1.04 | 1.04-1.05 | <0.001 |  | 1.04 | 1.03-1.05 | <0.001 |  | 1.04 | 1.035-1.05 | <0.001 |  | 1.04 | 1.03-1.05 | <0.001 |
| BMI | 1.01 | 0.99-1.03 | 0.451 |  | 1.01 | 0.99-1.03 | 0.405 |  | 0.996 | 0.97-1.02 | 0.727 |  | 0.99 | 0.96-1.01 | 0.331 |
| Physical activity (high vs. low) | 0.91 | 0.77-1.07 | 0.245 |  | 0.90 | 0.75-1.08 | 0.259 |  | 1.10 | 0.91-1.31 | 0.321 |  | 1.23 | 0.998-1.51 | 0.053 |
| Diabetes history | 1.11 | 0.53-2.33 | 0.790 |  | 1.13 | 0.62-2.07 | 0.683 |  | 1.13 | 0.61-2.06 | 0.703 |  | 0.96 | 0.49-1.89 | 0.906 |
| Smoking (ever) | 1.25 | 1.01-1.55 | 0.045 |  | 1.22 | 0.97-1.54 | 0.090 |  | 1.15 | 0.88-1.50 | 0.316 |  | 1.10 | 0.80-1.50 | 0.569 |
| Alcohol consumption (ever) | 1.27 | 1.04-1.54 | 0.018 |  | 1.22 | 0.99-1.50 | 0.057 |  | 1.26 | 0.997-1.59 | 0.053 |  | 1.14 | 0.88-1.50 | 0.324 |

The present COX regression model included all the covariates in the table above, and for categorical variable, such as diabetes history, we calculated the hazard ratio of future fracture in subjects with diabetes vs. without diabetes. BMI, body mass index.
